# Long-Read Draft Genome Sequences of Two *Fusarium oxysporum* f. sp. *cubense* Isolates from Banana (*Musa* spp.)

**DOI:** 10.3390/jof11060421

**Published:** 2025-05-30

**Authors:** Jiaman Sun, Jinzhong Zhang, Donald M. Gardiner, Peter van Dam, Gang Fu, Brett J. Ferguson, Elizabeth A. B. Aitken, Andrew Chen

**Affiliations:** 1 Guangdong Provincial Key Laboratory of Conservation and Precision Utilization of Characteristic Agricultural Resources in Mountainous Areas, School of Life Science, Jiaying University, Meizhou 514015, China; jiamansun@hotmail.com; 2 Guangxi Academy of Agricultural Sciences, Nanning 530007, China; fug110@gxaas.net; 3 Guangzhou Academy of Agricultural and Rural Sciences, Guangzhou 510335, China; jzzhang@foxmail.com; 4 Queensland Alliance for Agriculture and Food Innovation, The University of Queensland, St Lucia, QLD 4072, Australia; donald.gardiner@uq.edu.au; 5 Genetwister Technologies B.V., 6709 Wageningen, The Netherlands; 6 School of Agriculture and Food Sustainability, The University of Queensland, Brisbane, QLD 4072, Australia; b.ferguson1@uq.edu.au (B.J.F.); e.aitken@uq.edu.au (E.A.B.A.)

**Keywords:** Fusarium wilt of banana, PacBio HiFi, genome assembly, fungal effectors, pathogenicity, *Fusarium oxysporum* species complex, whole-genome phylogenetics

## Abstract

*Fusarium oxysporum* f. sp. *cubense* (*Foc*) causes Fusarium wilt, a devastating epidemic disease that has caused widespread damage to banana crops worldwide. We report the draft genomes of *Foc* race 1 (16117) and *Foc* tropical race 4 *(Fusarium odoratissimum)* (CNSD1) isolates from China, assembled using PacBio HiFi sequencing reads, with functional annotation performed. The strains group in distinct lineages within the *Fusarium oxysporum* species complex. This genetic resource will contribute towards understanding the pathogenicity and evolutionary dynamics of *Foc* populations in banana-growing regions around the world.

## 1. Introduction

Fusarium wilt of banana, caused by *Fusarium oxysporum* f. sp. *cubense* (*Foc*) is a devastating disease affecting banana production worldwide [1,2]. This disease, also known as Panama disease, occurs when *Foc* infects the vascular system of the banana plant, blocking water and nutrient flow, leading to plant wilting and eventual death [3]. The diversity within *Foc* populations can be differentiated using vegetative compatibility grouping (VCG), a classification system that shows association with virulence, host range, and geographic distribution of *Foc* populations [4]. Currently, at least 24 VCGs have been identified for *Foc*, underscoring the complexity and adaptability of this pathogen [5,6]. Alternatively, *Foc* can be classified into a race structure based on its virulence against specific banana cultivars it infects. *Foc* race 1 caused the pandemic that led to the demise of the ‘Gros Michel’ banana in the mid-20th century and also affects several other cultivars including ‘Lady Finger’ and ‘Silk’ bananas [3]. The virulent pathogen race, *Foc* tropical race 4 (TR4), has threatened global banana production due to the widespread reliance on Cavendish cultivars. The spread of *Foc* TR4 to Southeast Asia, the Middle East, Africa, and Latin America has caused serious concerns about its impact on banana production globally [7,8].

*Foc* TR4 and *Foc* race 1 both cause Fusarium wilt in banana but differ significantly in their host range, virulence, and impact on the banana industry. *Foc* race 1 caused the mid-20th-century collapse of ‘Gros Michel’ banana plantations but does not infect Cavendish cultivars, which subsequently became the dominant commercial variety. In contrast, *Foc* TR4 exhibits a broader host range, infecting all Cavendish bananas as well as those susceptible to *Foc* race 1. It is highly virulent, causing rapid plant decline and producing propagules that can persist in soil for up to a decade. The continued spread of *Foc* TR4 presents a major threat to global banana production, especially in monoculture-based export systems [2]. Understanding these differences is essential for developing diagnostic tools to aid management strategies in the control and deterrence of *Foc* TR4. To this end, two *Foc* isolates, confirmed as *Foc* race 1 and *Foc* TR4, were sequenced using PacBio long-read HiFi technology. These genome resources will contribute towards understanding differences in host specificity and virulence between these strains, thereby allowing improved strategies to manage *Foc* TR4 in the future.

## 2. Materials and Methods

### 2.1. Sample Collecting and Fungal Isolates

Banana pseudostem samples showing symptoms of Fusarium wilt were collected from fields in Baini, Foshan, Guangdong (23°2′48″ N, 112°52′50″ E) on dwarf banana plants and from fields in Wuming, Nanning, Guangxi, China (23°19′98″ N, 108°16′68″ E) on Cavendish banana. The pseudostem sections were surface-sterilized and cultured on potato dextrose agar (PDA) at 28°C for 3 days to isolate the fungus. Single spore isolation was then performed to generate monoconidial cultures for each of these isolates. Prior to this study, vegetative compatibility group (VCG) testing confirmed the identities of both isolate 16117 (Baini) and CNSD1 (Wuming) as *Foc* race 1 (VCGs 0120/15, 01218) and *Foc* TR4 (VCG 01213/16), respectively. The *Foc* TR4 isolate CNSD1 was used in a previous study to assess transcriptome reprogramming in response to *Foc* TR4 in resistant and susceptible banana cultivars [9].

### 2.2. DNA Extraction and Genome Sequencing

Monoconidial cultures of isolates 16117 and CNSD1 were incubated on PDA medium for 7 days and mycelia were scraped off the plates and used for DNA extraction. Genomic DNA of the two isolates were then extracted using the MagAttract HMW DNA kit (Qiagen, Hilden, Germany) according to manufacturer’s instructions, and the purified DNA was quantified with a Qubit (4.0) fluorimeter (Life Technologies, Carlsbad, CA, USA). The isolates were sequenced on a PacBio SMRT flow cell (Menlo Park, CA, USA). Libraries were prepared by Personalbio Technology Co., Ltd. (Shanghai, China) using a standard PacBio gDNA library preparation kit. Briefly, the high molecular weight genomic DNA was sheared by g-TUBE to an average size between 10 and 15 Kb, ligated with known adapters and digested by an enzyme reaction. The BluePippin (Sage Science, Beverley, MA, USA) was used to select the DNA fragment with a target size of 10–15 Kb to generate SMRTbell structure libraries. The purified libraries were analyzed by an Agilent 2100 Bioanalyzer System (Agilent technologies, Santa Clara, CA, USA) before sequencing on the PacBio Sequel II platform.

### 2.3. Genome Assembly and Gene Prediction

The resulting long reads were subject to de novo assembly using Hifiasm v0.18.5 under the Galaxy Australia compute environment (Version 0.24.0), with default settings including -k 51 and -l0 [10]. The assemblies were initially evaluated using QUAST v5.3.0 [11]. The genome assembly completeness was further assessed using BUSCO v5.4.5, with AUGUSTUS v3.4.0 configured with the *Fusarium graminearum* species training set against the Ascomycota lineage [12].

The assemblies were then annotated for elements, including repeat sequences and protein-coding genes. Repeats in the genomes were identified using RepeatModeler (v2.0.4) and then masked using RepeatMasker (v4.1.4) software [13]. Protein-coding gene prediction was performed by GlimmerHMM (v3.0.4) [14], AUGUSTUS (version 2.5.5) [15], and GeneMark-ES (v4.71) software [16]. To refine gene annotations, additional gene structure evidence was obtained by performing homology-dependent alignments to five *F. oxysporum* genomes using exonerate (v2.2.0) [17]. These included *F. oxysporum* Fo47 (GCF_013085055.1), *F. oxysporum* f. sp. *lycopersici* MN25 (GCA_000259975.2), *F. oxysporum* f. sp. *pisi* HDV247 (GCA_000260075.2), *F. oxysporum* f. sp. *radicis-lycopersici* 26381 (GCA_000260155.3), and *F. oxysporum* f. sp. *vasinfectum* 25433 (GCA_000260175.2). The predicted gene models were then integrated into a weighted consensus gene set using EvidenceModeler v 2.0.0 [18] to generate a final high-confidence gene annotation set.

The mitochondrial genomes of both isolates were assembled using MitoHiFi version 3 [19] and annotated using the mitochondrial genome of *Fusarium oxysporum* strain 19–385 (NCBI: OR601176) as a reference in the Galaxy Australia computing environment (usegalaxy.org.au) [20]. MitoHiFiv3.2.3 used the contig assembly (-c), along with a reference mitochondrial genome and its GenBank-formatted annotation, as input. Mitochondrial contigs were first assembled using Hifiasm and then filtered for sequence homology by comparing them to the reference genome with NCBI BLAST+. Candidate contigs were subsequently annotated with MitoFinder v1.4.0, which performed BLAST similarity searches against the reference nucleotide and protein sequences [19].

### 2.4. Chromosome Alignments

Telomere repeats of 5′-TAACCC-′3 were first detected using the search function of tidk (version 0.2.63) and then visualized using its plot function [21].

The assembly scaffolds were aligned with chromosome-level reference genomes using the nucmer function of MUMmer software (version 4.0.1), with default settings [22]. An interactive web plot viewer Dot (https://github.com/marianattestad/dot, accessed on 12 February 2025) was then used to visualize an ordered set of reference and query alignments, passing in the nucmer outputs using the script DotPrep.py, and applying filtering to display aligned regions of greater than 2 Kb.

### 2.5. Functional Annotation

Genes were functionally annotated by performing searches against multiple databases. Specifically, genes were searched in BLAST against the non-redundant protein database to identify homologous proteins [23]. GO annotation was performed using InterPro (version 66.0, release 2017.11.23) [24]. The results were then processed in InterPro2GO to obtain GO terms, which were then mapped to a list of selected terms (GO slims) using map2slim (https://github.com/elhumble/map2slim, accessed on 18 February 2025). Database searches using eggNOG (http://eggnogdb.embl.de/) and an E-value threshold of 1 × 10^−6^ to infer orthologous groups and functional annotations, the KEGG database (https://www.kegg.jp/) to associate genes with metabolic and signaling pathways, and the CAZy database (http://www.cazy.org/) to classify carbohydrate-active enzymes were performed using Diamond (v2.0.14) [25].

To further characterize protein function, localization signals, including signal peptides, were predicted from the draft genomes of strains 16117 and CNSD1 using SignalP (v5.0) [26] and TargetP (v2.0) [27]. Membrane protein topology was predicted using TMHMM version 2.0 [28]. Additionally, potential secreted effectors, proteins that may play a role in host–pathogen interactions, were predicted using EffectorP (v3.0) [29].

### 2.6. Phylogenetic Analysis

The phylogenetic analysis was performed using the publicly available genomes of 152 *F. oxysporum* strains, including special forms on banana [30,31,32] and other plant hosts, as well as a *F. verticillioides* (isolate 7600) strain, used to anchor the whole phylogeny. The isolates derived from banana, along with their corresponding genome accession numbers from previous studies, are listed in Table S1. All other *F. oxysporum* genomes were obtained directly from NCBI. Accession numbers for all genomes included in the phylogenetic analysis are embedded in their names within the phylogeny. The retrieval of the assemblies, AUGUSTUS annotation, and the subsequent phylogenetic analysis were performed using a workflow described in another study [33].

Briefly, all genomes were loaded into the Galaxy web platform via the public server at usegalaxy.org.au to analyze the data [20]. De novo gene annotation was performed using AUGUSTUS (version 3.4.0) [15,34,35], with *Fusarium graminearum* splice models. Only genes without internal stop codons were retained. BUSCO was then performed on these coding sequences to retain only complete, single-copy conserved genes [12], using a custom bash script. Single-copy genes across all 152 genomes were identified using seqkit grep (version 2.9.0) [36] and aligned using MAFFT (version 7.520) [37] with default settings, and poorly aligned regions were removed using trimAI (version v1.5.rev0) [38]. The final edited alignments were concatenated using the seqkitconcat command.

Phylogenetic reconstruction based on DNA sequences was conducted using RAxML GUI (version 2.0) [39]. The best-fit model, GTR + I + G4, was selected and applied for maximum likelihood tree inference, with 100 bootstrap replicates performed to assess branch support. *F. verticillioides* isolate 7600 was designated as the outgroup. The resulting phylogenetic tree was then imported into the Interactive Tree of Life (iTOL) v7 [40].

### 2.7. Genome-Wide Profiling of SIXGene Effectors

The *Fusarium oxysporum* Effector Clustering (FoEC2) pipeline was run using the *Foc* genomes retrieved from the public databases and the ones obtained in this study [41]. Fourteen *SIX* gene nucleotide sequences previously obtained from a *Fol* strain were also used as a query [42]. Clustering in the pipeline was performed using default settings which included binary distance matrix and average distance calculation. A TBLASTN search of Fol-SIX protein sequences against all *Foc* genomes was performed using the command line version of NCBI-BLAST+ (version v2.12.0) and an e-value cut-off score of 1 × 10^−10^.

## 3. Results and Discussion

### 3.1. Pathogen Isolation

**Figure 1:**
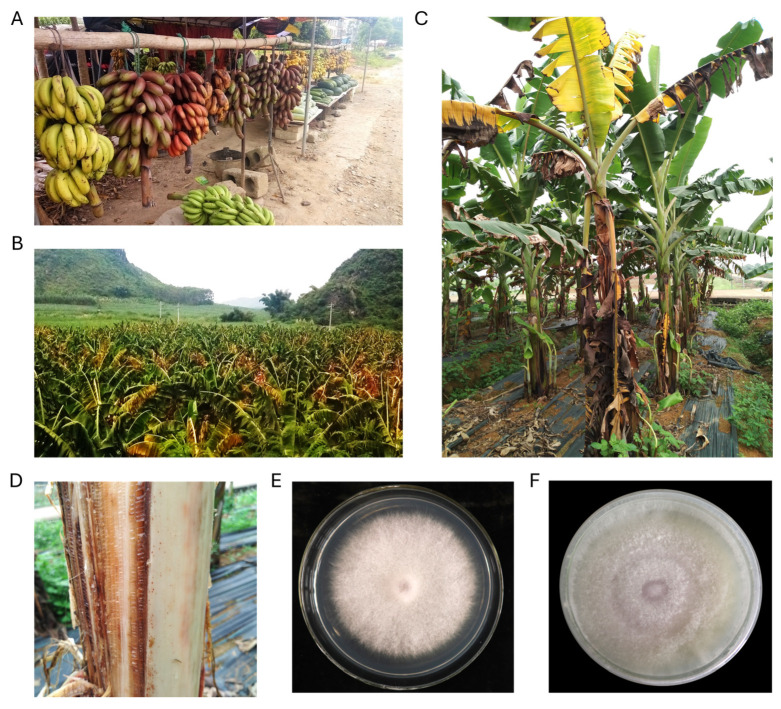
Local banana production regions in Guangxi, China. (**A**) A local farm holder selling freshly produced bananas from the ‘AAA’ cultivar group, including *Musa acuminata* ‘Red Dacca’ and ‘Williams’ Cavendish in Wuming, Nanning, Guangxi, China. (**B**) Field-grown symptomatic banana plants infected with Fusarium wilt in Wuming, Nanning, Guangxi, China, where *Fusarium oxysporum* f. sp. *cubense* isolate CNSD1 was collected. (**C**) An individual banana plant showing Fusarium wilt symptoms in Wuming. (**D**) Internal discoloration observed in the pseudostem of this banana plant. *Fusarium oxysporum* f. sp. *cubense* monoconidial isolates (**E**) 16117 and (**F**) CNSD1 grown on potato dextrose agar.

Fusarium wilt caused by *Foc* TR4 has placed a heavy burden on local farm holders who rely on fresh locally grown bananas as a primary source of income in Wuming, Guangxi, China (Figure 1A). *Foc* TR4 isolate CNSD1 was isolated from plants in a local banana plantation in Wuming, which exhibited severe Fusarium wilt symptoms, including leaf yellowing, necrosis, and vascular wilt of entire plants (Figure 1B–D). *Foc* race 1 isolate 16117 was isolated from a dwarf banana plant in Baini, Foshan, Guangdong. After single spore isolation, both isolates showed a light pink color, with aerial hyphae observed, when grown on PDA (Figure 1E,F).

### 3.2. Mitochondrial Genomes

The mitochondrial genomes of 16117 and CNSD1 were 45,628 bp and 49,694 bp, respectively (Figure S1). Analysis of *Fusarium oxysporum* mitochondrial genomes suggests the presence of a large variable region, which comes in the form of three distinct haplotypes or variants [43]. The mitochondrial genomes of 16117 and CNSD1 were similar to that of *Fusarium oxysporum* f. sp. *cubense* race 4 strain B2 (LT571433), carrying the haplotype as large variable region 1 [43].

### 3.3. Nuclear Genomes

**Figure 2:**
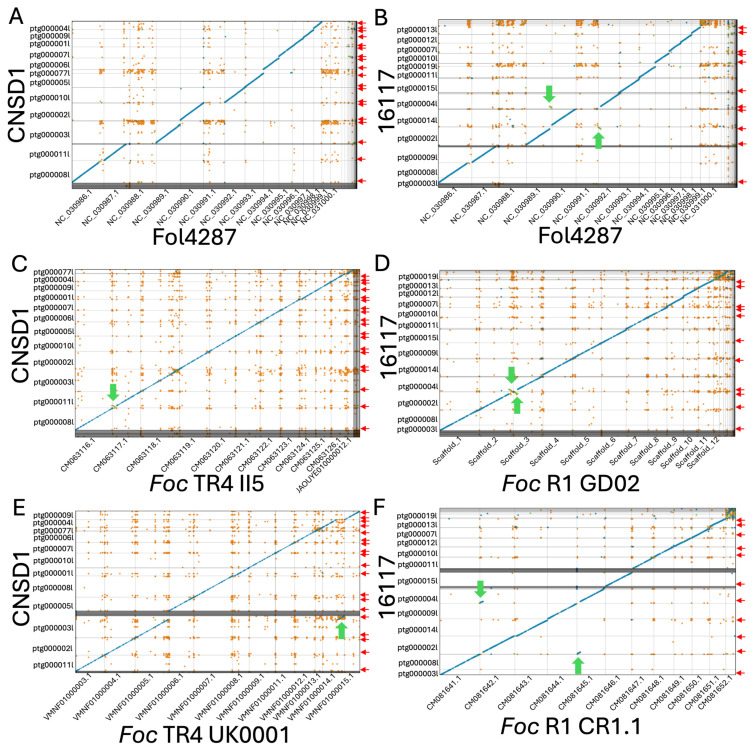
Whole-genome contig alignment of *Fusarium oxysporum* f. sp. *cubense* TR4 isolate CNSD1 (**A**) and race 1 isolate 16117 (**B**) to each of the 15 chromosomes of the *F. oxysporum* f. sp. *lycopersici* strain 4287 (Fol4287) genome. (**C**) Contig alignment of CNSD1 to *Foc* TR4 II5. (**D**) Contig alignment of 16117 to *Foc* R1 GD02. (**E**) Contig alignment of CNSD1 to *Foc* TR4 UK0001. (**F**) Contig alignment of 16117 to *Foc* R1 CR1.1. Unique alignments are shown in blue (forward) and green (reverse complement). Repetitive alignments are shown in orange. Only alignments greater than 2 Kb are shown. Red arrows indicate the presence of telomere repeats detected at the terminal ends of the contigs. Green arrows indicate the observed chromosomal rearrangements between a pair of chromosomes.

Nuclear genomes of both isolates were assembled into primary contigs with N50s of 4.5 Mbp (16117) and 4.2 Mbp (CNSD1), resembling the size of entire chromosomes in *Fusarium oxysporum* species (Table 1). Genome completeness analysis using BUSCO showed that both genomes encoded near-complete sets of conserved genes (Table 1). A total of 19 and 11 telomere regions containing the 5′-TAACCC-3′ repeats were identified on the contigs corresponding to the 11 core chromosomes of CNSD1 and 16117, respectively (Figure 2 and Figures S2 and S3).

Both isolates appeared to have the equivalents for 11 of the 15 chromosomes in *F. oxysporum* f. sp. *lycopersici* (*Fol*) strain 4287, while lacking the equivalents of *Fol* chromosomes 3, 6, 14, and 15 (Figure 2A,B). The accessory sequences of these genomes did not align with any chromosomes in *Fol* strain 4287. When aligned to a near-complete genome of *Foc* TR4 isolate II-5 and *Foc* race 1 isolate GD02 derived from a previous study [31], all 11 core chromosomes of *Foc* strains 16117 and CNSD1 aligned well to their counterparts (Figure 2C,D). The accessory sequence from CNSD1 also aligned well to its counterpart in II-5 (Figure 2C). In the race 1 comparison, these sequences appeared fragmented and variable in size (Figure 2D). This accessory sequence has been examined in terms of its structural variation and gene content in several studies [30,31].

### 3.4. Chromosome Rearrangements

When aligned to the *Foc* TR4 II-5 and UK0001 genomes, CNSD1 showed a segmental inversion in contig ptg000011l that is present only in the *Foc* TR4 II-5 genome (Figure 2C,E). *Foc* TR4 UK0001 also harbors a chromosome segmental translocation identified in an earlier study [31]. In *Foc* race 1 assembly 16117, a putative reciprocal translocation event was detected in contigs ptg000002l and ptg000004l, when it was aligned to *Foc* race 1 genomes GD02 and CR1.1 (Figure 2D,F). Telomeric repeats were also identified on the ends of all three contigs involved in these rearrangements. The assemblies will have to be validated by other means to confirm whether these rearrangements are genuine events or assembly errors.

### 3.5. Functional Annotations

A total of 15,943 and 15,247 protein-coding genes were annotated in 16117 and CNSD1, respectively (Table 1, Additional Files S1 and S2). GO classification for these two genomes included an abundance of terms associated with carbohydrate, lipid, nitrogen metabolism, cell wall biogenesis, and enzymes and transport activities (Figures S4 and S5, Additional Files S3 and S4). This is also evident in the representative groups (carbohydrate, lipid, and amino acid) within metabolism and cellular processes identified in the KEGG pathway classifications for each isolate (Figures S6 and S7, Additional Files S5 and S6). For both isolates, the three most abundant KOG categories were carbohydrate transport and metabolism, secondary metabolites biosynthesis, transport and catabolism, and amino acid transport and metabolism (Tables S2 and S3, Additional Files S7 and S8).

CAZymes (Carbohydrate-active enzymes) are a diverse group of enzymes that play key roles in the breakdown, modification, and synthesis of carbohydrates. Some of these enzymes can be considered components of pathogen-secreted proteins involved in infection processes. Classification into the six CAZyme subfamilies [44] revealed that both isolates contained approximately 900 carbohydrate-active enzymes, with glycoside hydrolases being the most abundant, numbering over 300 (Tables S4 and S5, Additional Files S9 and S10).

### 3.6. Phylogenetic Analysis

**Figure 3:**
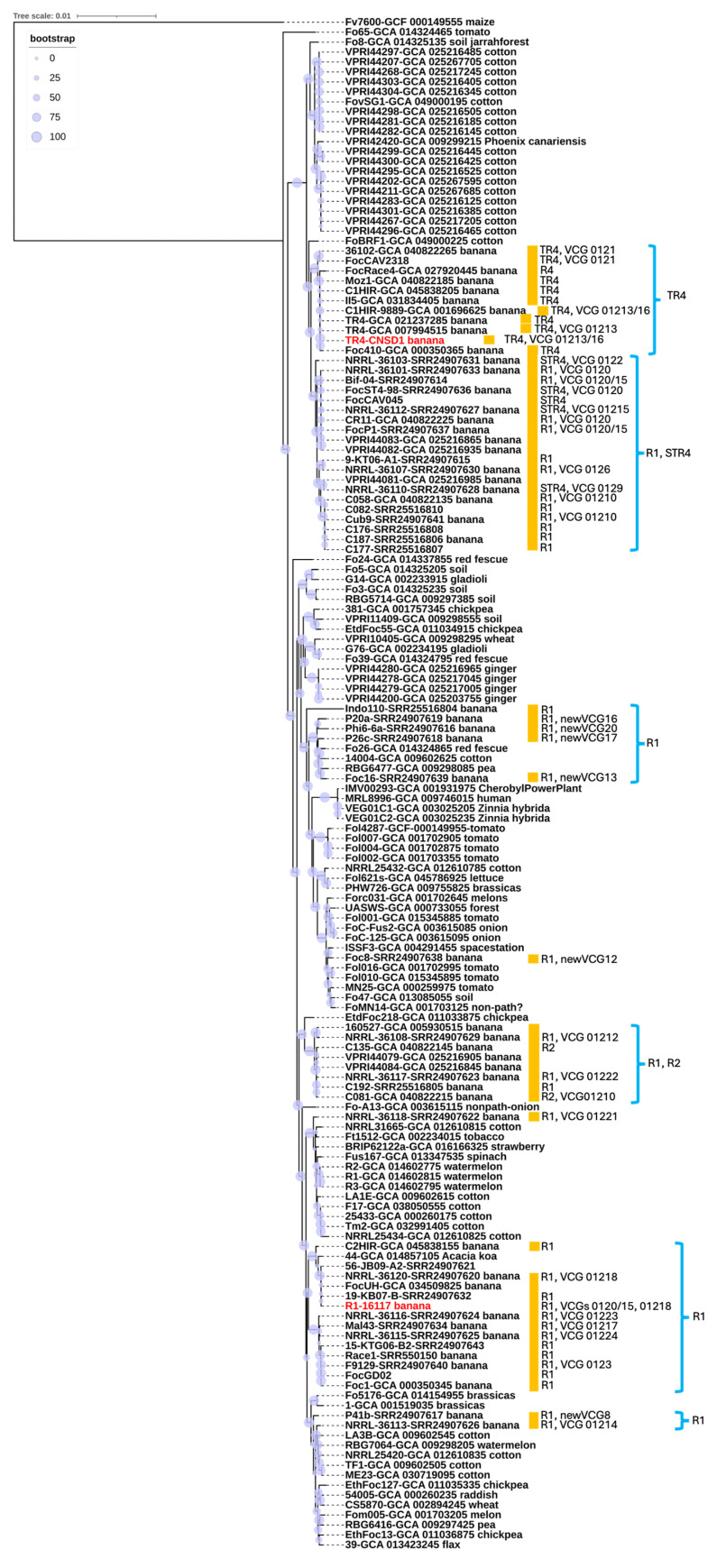
Whole genome-based phylogenetic reconstruction based on a BUSCO set of 894 conserved single-copy orthologs shared among 154 representatives of the *Fusarium oxysporum* species complex. Most *Foc* genomes and VCG information were retrieved from previous studies [30,31]. R1, R2, STR4, and TR4 annotate race 1, race 2, subtropical race 4, and tropical race 4, respectively. Red highlight indicates the strains sequenced in this study. The host of origin is indicated, along with the race and VCG designations for banana isolates (marked with orange boxes) where this information could be found in NCBI databases or publications. Branch support based on bootstrap analysis (percentage) is indicated by circles scaled relative to a 0–100% bootstrap value.

A phylogenetic tree was constructed using a total of 154 genomes, including the two newly sequenced in this study. The multiple sequence alignment analysis encompassed 1.49 × 10^6^ nucleotide sites, with nearly 10% of the positions exhibiting variation across the alignment, highlighting substantial genetic diversity within the dataset. *F. oxysporum* f. sp. *cubense* isolate CNSD1 clustered together with other *Foc* TR4 isolates in a TR4-specific phylogroup (Figure 3). The phylogenetic placement of *Foc* TR4 isolates supports their distinction from *Foc* STR4 and race 1 isolates, consistent with findings from previous studies [45,46]. The *Foc* TR4 phylogroup exhibited little genetic variations among its members (Figure 3), which is also evident from a previous study [47]. In contrast, *F. oxysporum* f. sp. *cubense* isolate 16117 grouped with three other *Foc* isolates, two of which are classified as race 1. The positioning of this phylogroup was distinct from other clusters containing *Foc* race 1 strains, suggesting an independent evolutionary origin. This finding underscores the genomic diversity of *Foc* race 1 isolates, consistent with previous reports [45]. Overall, the phylogenetic analysis supports the polyphyletic nature of *Fusarium oxysporum* strains isolated from banana plants. The presence of multiple, genetically distinct lineages within the *F. oxysporum* species complex suggests that these banana-derived strains may have evolved independently. This pattern could potentially be influenced by horizontal gene transfer [46]. However, without functional evidence confirming host specificity, such as pathogenicity assays or comparative genomic analysis of host-determining regions, caution is needed in interpreting these lineages as independently evolved banana pathogens. These findings reinforce the complex evolutionary dynamics underlying the genetic diversity of *F. oxysporum* strains associated with banana, highlighting the need for further investigation into their pathogenic potential and host range.

### 3.7. Effector Annotation

Fungal effectors are small, secreted proteins that aid fungal pathogens in infecting their hosts by suppressing host immune responses. These effectors are typically characterized by their small size and a high cysteine content, which contributes to stability.

To define the secretomes of *F. oxysporum* f. sp. *cubense* isolates 16117 and CNSD1, proteins carrying signal peptides were independently identified using the prediction programs SignalP and TargetP. A total of 1357 and 1270 genes encoding secreted proteins were identified for isolates 16117 and CNSD1, respectively, after excluding those containing transmembrane domains (Table S6) (Additional Files S9 and S10).

Effector profiling with EffectorP predicted a total of 420 and 417 apoplastic effectors within the genomes of isolates 16117 and CNSD1, respectively (Additional Files S11 and S12). Of these, 265 effectors in isolate 16117 and 256 in isolate CNSD1 were found to be secreted proteins containing signal peptides, as determined by comparison against each isolate’s respective secretome. These numbers are consistent with effector predictions previously reported for other *Fusarium oxysporum* isolates and *Fusarium* species [29,48]. The identification of these effectors will provide valuable insights into the molecular mechanisms driving their pathogenicity in banana.

### 3.8. SIX Gene Profiles of Fusarium oxysporum f. sp. cubense Genomes

All *SIX* gene homologs identified based on an E-value threshold of 1 × 10⁻^10^ had a minimum amino acid identity of 45%. The majority of the hits had a query coverage of ≥70% with hits lower than 70% (grey highlight) mainly associated with duplicated copies or regions of *SIX1*, *SIX4*, *SIX8*, and *SIX13* (Additional File S13).The genome of CNSD1 encoded homologs for *SIX1*, *SIX2*, *SIX4*, *SIX6*, *SIX8*, *SIX9*, and *SIX13*, whereas the 16117 genome appeared to carry homologs for only *SIX1*, *SIX9*, and *SIX13* (Table S7). In CNSD1, multiple copies of *SIX* genes, including three copies of *SIX1*, two copies of *SIX8*, three copies of *SIX9*, and two copies of *SIX13*, were detected. Out of the 13 *SIX* gene homologs detected in CNSD1, 10 were located on contig ptg000003l. Isolate 16117, on the other hand, carried two copies of *SIX1*, three copies of *SIX9*, and two copies of *SIX13* (Table S7).

**Figure 4:**
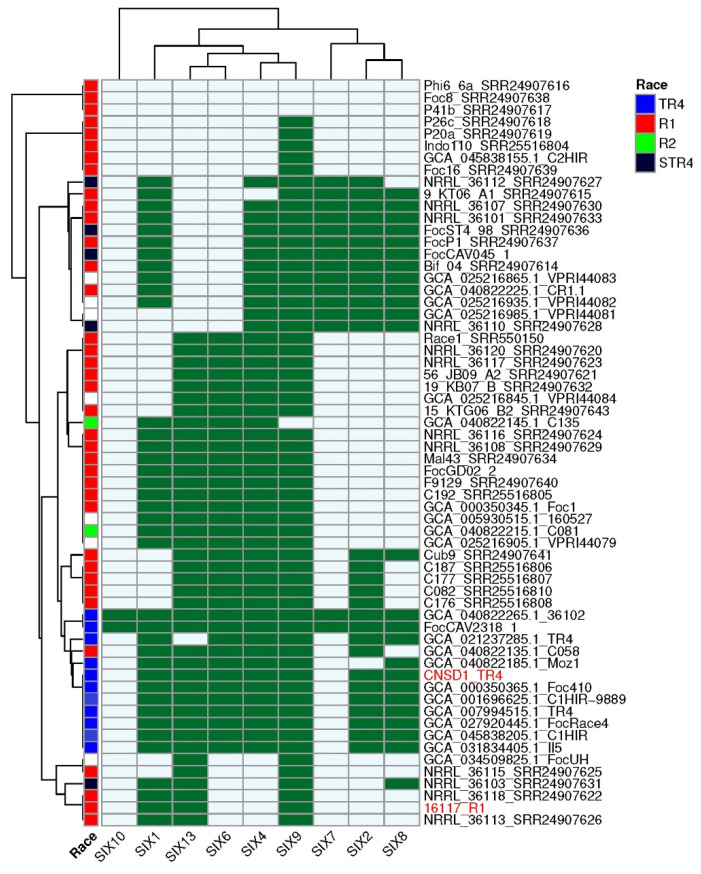
*SIX* gene profiles of 62 *Fusarium oxysporum* f. sp. *cubense* genomes determined using the FoEC2 pipeline. *Foc* race designation for each isolate is provided where available [30,31]. Dark green and light blue boxes indicate the presence and absence of a *SIX* gene homolog, respectively. *SIX* homologs that are absent in this collection (*SIX3*, *SIX5*, *SIX11*, *SIX12*, and *SIX14*) are not shown. Red highlight indicates the strains sequenced in this study.

Analysis of the presence and absence of *SIX* genes across all 62 *Foc* genomes revealed that CNSD1 clustered with the other known *Foc* TR4 genomes, consistent with both the number of *SIX* genes typically present in TR4 genomes and the position of this phylogroup in the phylogenetic tree derived from conserved genes (Figure 4). TR4 strains FocCAV2318_1 and 36102 have additional homologs corresponding to *SIX7* and *SIX10* genes that are otherwise absent in all other TR4 strains. Isolate C058 clustered within the TR4 phylogroup based on its *SIX* gene profile, despite having been designated as a race 1 isolate.

The presence and absence of *SIX* genes in the race 1 strains confirmed their polyphyletic nature, with multiple phylogroups being apparent (Figure 4). Phylogroups that lacked any *SIX* genes (Phi6_6a, Foc8, and P41b), those possessing a single *SIX* gene (P26a, P20a, Indo110, C2HIR and Foc16), and those grouping with 16117 possessing two to four *SIX* genes (NRRL_36115, NRRL_36103, NRRL_36118, NRRL_36113) are incongruent with their positions in the phylogenetic tree derived from conserved genes (Figure 3 and Figure 4). However, the larger race 1 phylogroups that contained four to six *SIX* genes and interspersed with either race 2 or STR4 strains were largely congruent with the conserved gene phylogeny.

## 4. Conclusions

The fungal genome assemblies presented in this study are essential for managing Fusarium wilt in bananas. They provide valuable insights into the pathogenic mechanisms of *Foc* TR4 and race 1, their evolutionary origins, and banana–*Fusarium* interactions, all of which will aid in the development of resistant banana cultivars. Additionally, these resources support the improvement of disease diagnostics and the formulation of sustainable strategies to combat Fusarium wilt in banana.

## Figures and Tables

**Table 1:** Assembly statistics for *Fusarium oxysporum* f. sp. *cubense* isolate 16117 and CNSD1.

Statistics	16117 (Race 1)	CNSD1 (TR4)
Assembly		
Total sequence data (Gbp)	8.46	10.5
Coverage (fold)	170	223
Assembly size (bp)	51,695,064	49,684,144
No. of contigs	92	77
Largest contig (bp)	5,598,307	6,666,412
N50 contig length (bp)	4,227,447	4,512,489
Contig L50	6	5
Contig L90	14	11
GC content (%)	47.87	47.72
BUSCO coverage (%)	98.4	98.4
Total no. of BUSCOs	1706	1706
No. of duplicated BUSCOs	9	6
No. of fragmented BUSCOs	8	8
No. of missing BUSCOs	19	20
Gene models		
Total no. of protein-coding genes	15,943	15,247

## Data Availability

The original raw data presented in the study are openly available in the NCBI Sequence Read Archive (SRA) under accession numbers SRR31177457 (16117) and SRR31177522 (CNSD1). The genome assemblies described in this study are available in GenBank under Bioproject accession numbers PRJNA1178358 for strain 16117 and PRJNA1174872 for CNSD1. The data analysis outputs presented in this study are included in the Supplementary Material/additional files.

## Supplementary Materials

The following supporting information can be downloaded at: https://www.mdpi.com/article/10.3390/jof11060421/s1, Figure S1: Mitochondrial genomes of isolates 16117 (A) and CNSD1 (B); Figure S2: Enrichment of 5′-TAACCC-3′ repeats detected at the telomeres of contigs corresponding to the 11 core chromosomes of CNSD1 using Tidk; Figure S3: Enrichment of 5′-TAACCC-3′ repeats detected at the telomeres of contigs corresponding to the 11 core chromosomes of 16117 using Tidk; Figure S4: GO term classifications for the genome of isolate 16117; Figure S5: GO term classifications for the genome of isolate CNSD1; Figure S6: KEGG classifications for the genome of isolate 16117; Figure S7: KEGG classifications for the genome of isolate CNSD1; Table S1: Banana-derived isolates and their associated genome data or assemblies used in this study; Table S2: Clustering of *Fusarium oxysporum* f. sp. *cubense* isolate 16117 proteins based on the functional classification of KOG; Table S3: Clustering of *Fusarium oxysporum* f. sp. *cubense* isolate CNSD1 proteins based on the functional classification of KOG; Table S4: Classification of *Fusarium oxysporum* f. sp. *cubense* isolate 16117 proteins into the six CAZyme subfamilies; Table S5: Classification of *Fusarium oxysporum* f. sp. *cubense* isolate CNSD1 proteins into the six CAZyme subfamilies; Table S6: Number of effectors in the genomes of *Fusarium oxysporum* f. sp. *cubense* isolates 16117 and CNSD1; Table S7: SIX gene homologs detected in the genomes of CNSD1 and 16117 using TBLASTN with Fol-Six protein sequences used as queries. File S1: CNSD1.gff3; File S2: 16117.gff3; File S3: CNSD1 GO_annotation.xls; File S4: 16117 GO_annotation.xls; File S5: CNSD1_kegg.xls; File S6: 16117_kegg.xls; File S7: CNSD1_KOG_annotations.xls; File S8: 16117_KOG_annotations.xls; File S9: CNSD1_cazy_anno.xls; File S10: 16117_cazy_anno.xls; File S11: 16117_effector.xls; File S12: CNSD1_effector.xls; File S13: TBLASTN-SIX-genes.xlsx.
