# Supplementary material for: Long-Read Draft Genome Sequences of Two Fusarium oxysporum f. sp. cubense Isolates from Banana (Musa spp.)
Source: J Fungi (Basel). 2025 May 30;11(6):421. doi: 10.3390/jof11060421 (PMC12193908; doi:10.3390/jof11060421)
Supplement: Supplementary file 1 [file jof-11-00421-s001.zip › Supplemental file.pdf]

Communication

Long-Read Draft Genome Sequences of Two *Fusarium oxysporum* f. sp. *cubense* Isolates from Banana (*Musa* spp.)

Authors

Jiaman Sun, Jinzhong Zhang, Donald M. Gardiner, Peter van Dam, Gang Fu, Brett J. Ferguson, Elizabeth A. B. Aitken and Andrew Chen

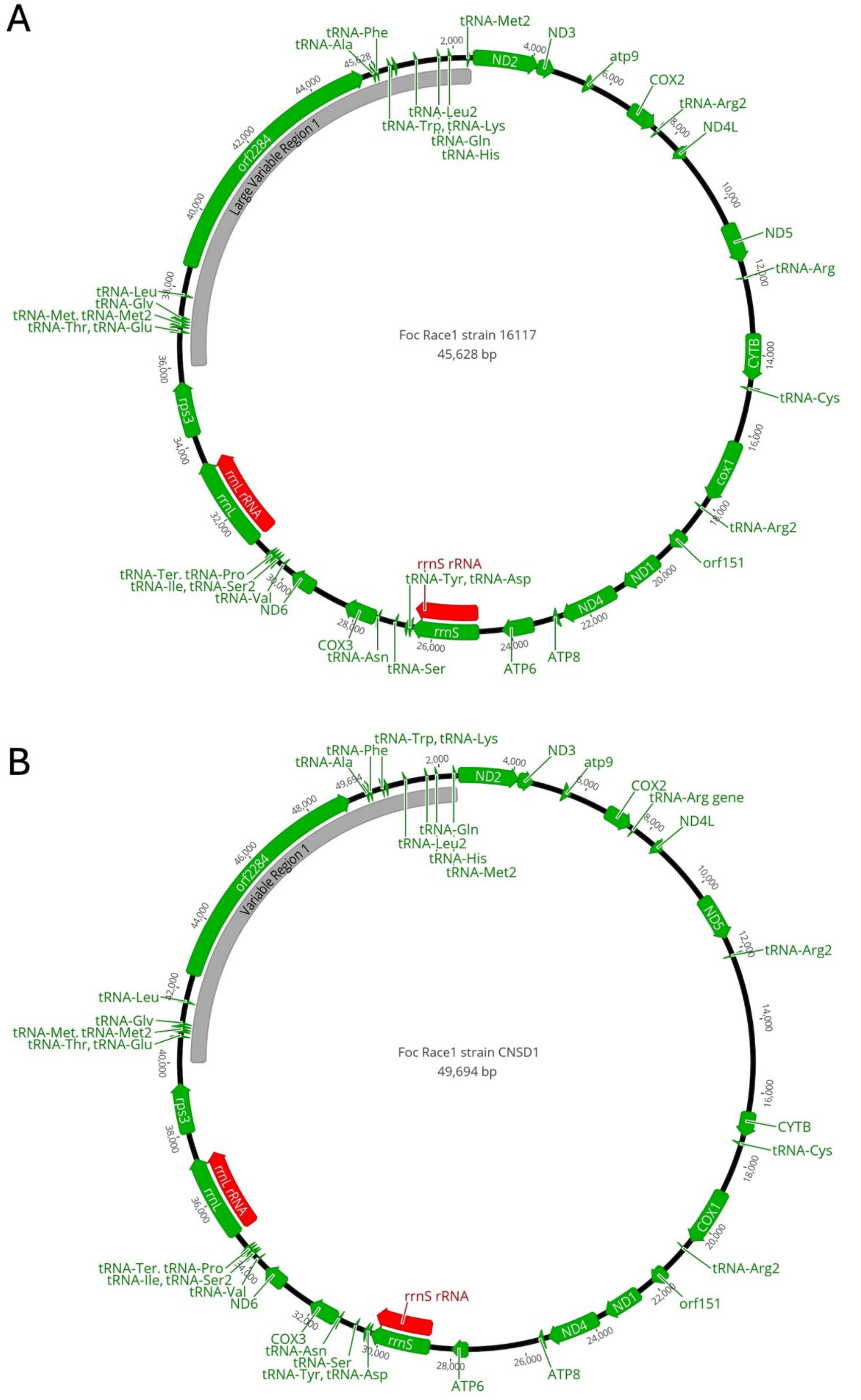

Supplemental Figure S1. Mitochondrial genomes of isolates 16117 (**A**) and CNSD1 (**B**). Genes were annotated by MitoHiFi using the reference mitochrondrial genome from the *Fusarium oxysporum* strain 19-385. Ribosomal RNA sequences are highlighted in red. The variable region 1 sequence is annotated grey in each sequence.

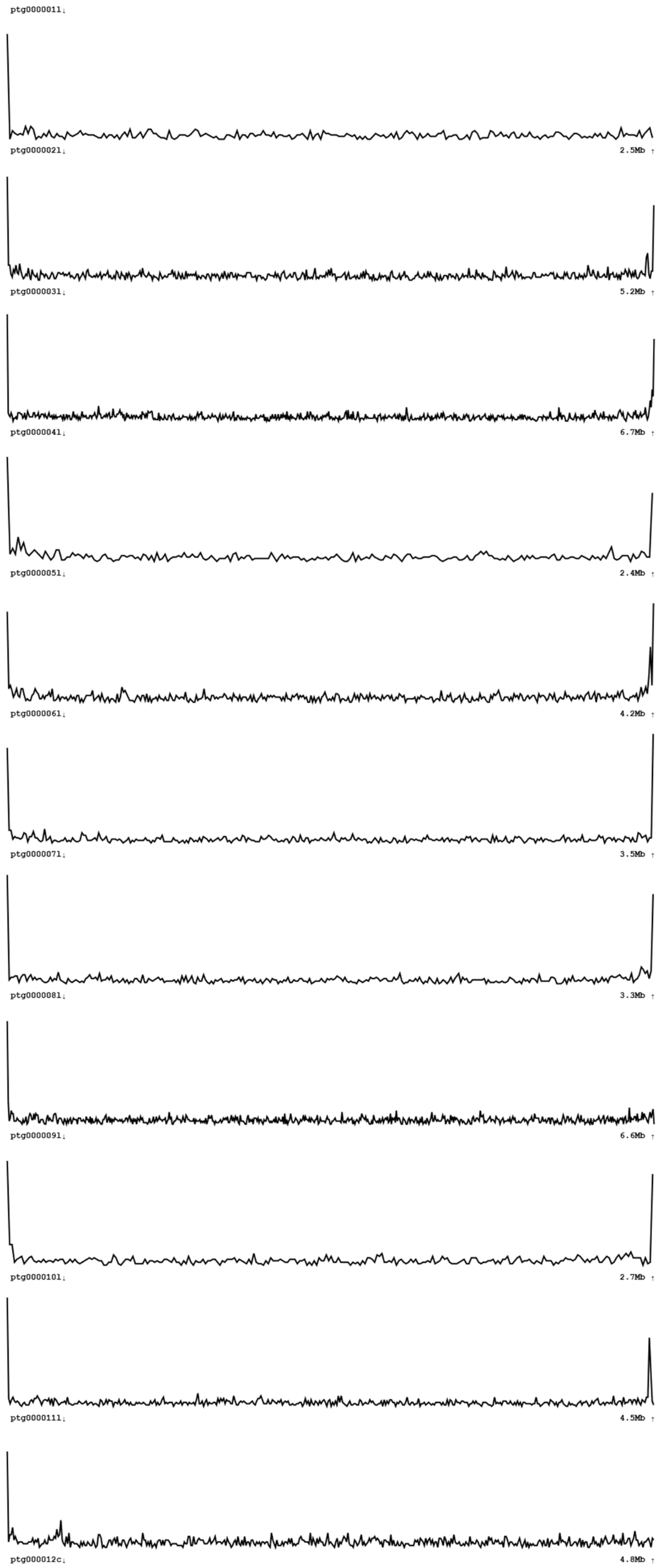

Supplemental Figure S2. Enrichment of 5'-TAACCC-3' repeats detected at the telomeres of contigs corresponding to the 11 core chromosomes of CNSD1 using Tidk.

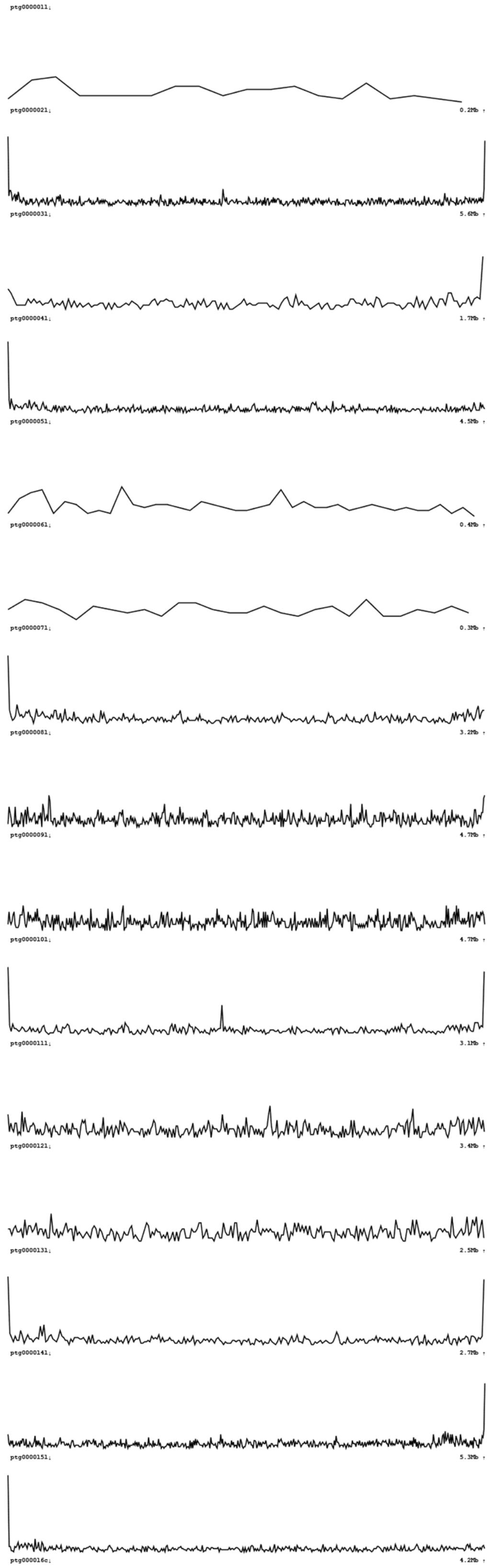

Supplemental Figure S3. Enrichment of 5'-TAACCC-3' repeats detected at the telomeres of contigs corresponding to the 11 core chromosomes of 16117 using Tidk.

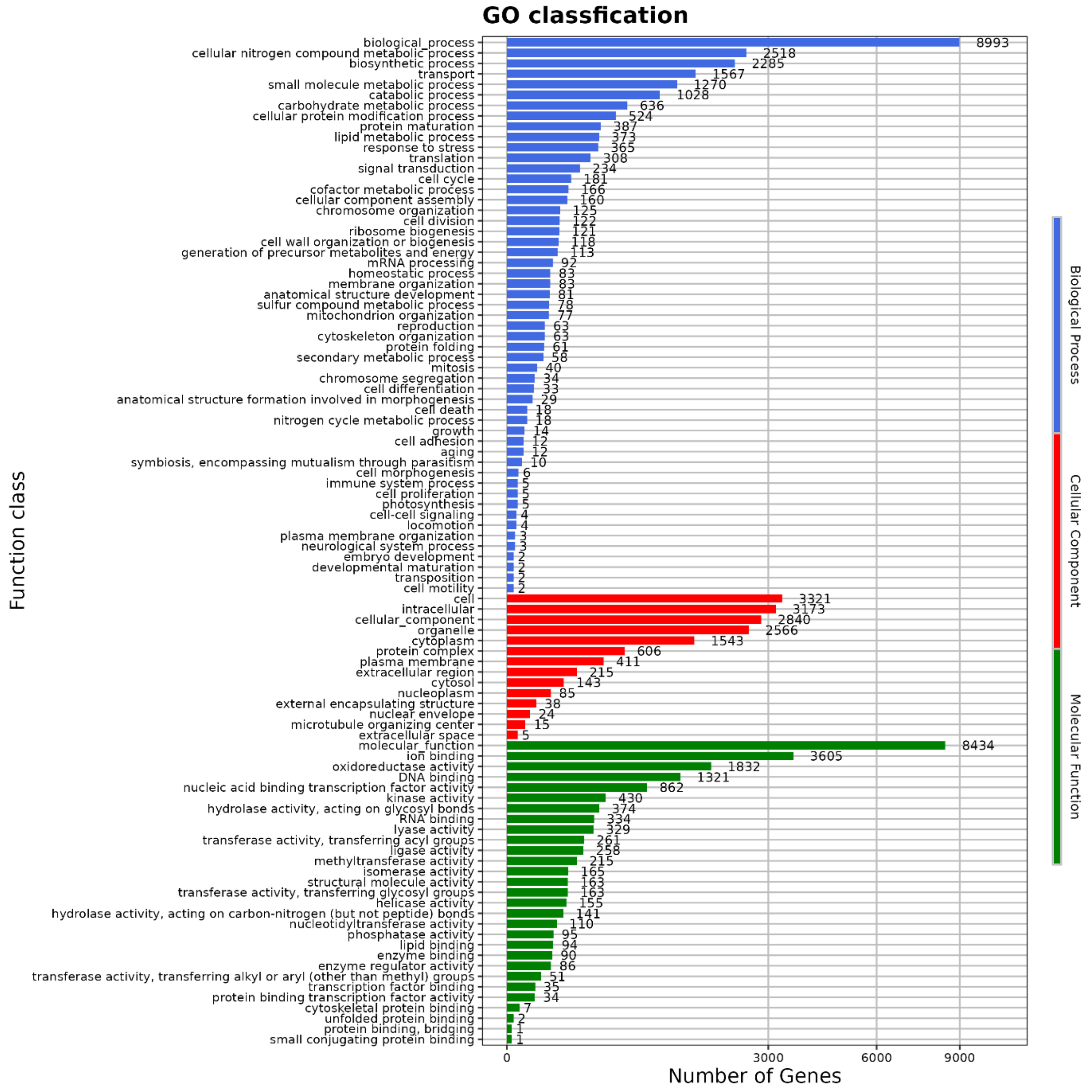

Supplemental Figure S4. GO term classifications for the genome of isolate 16117.

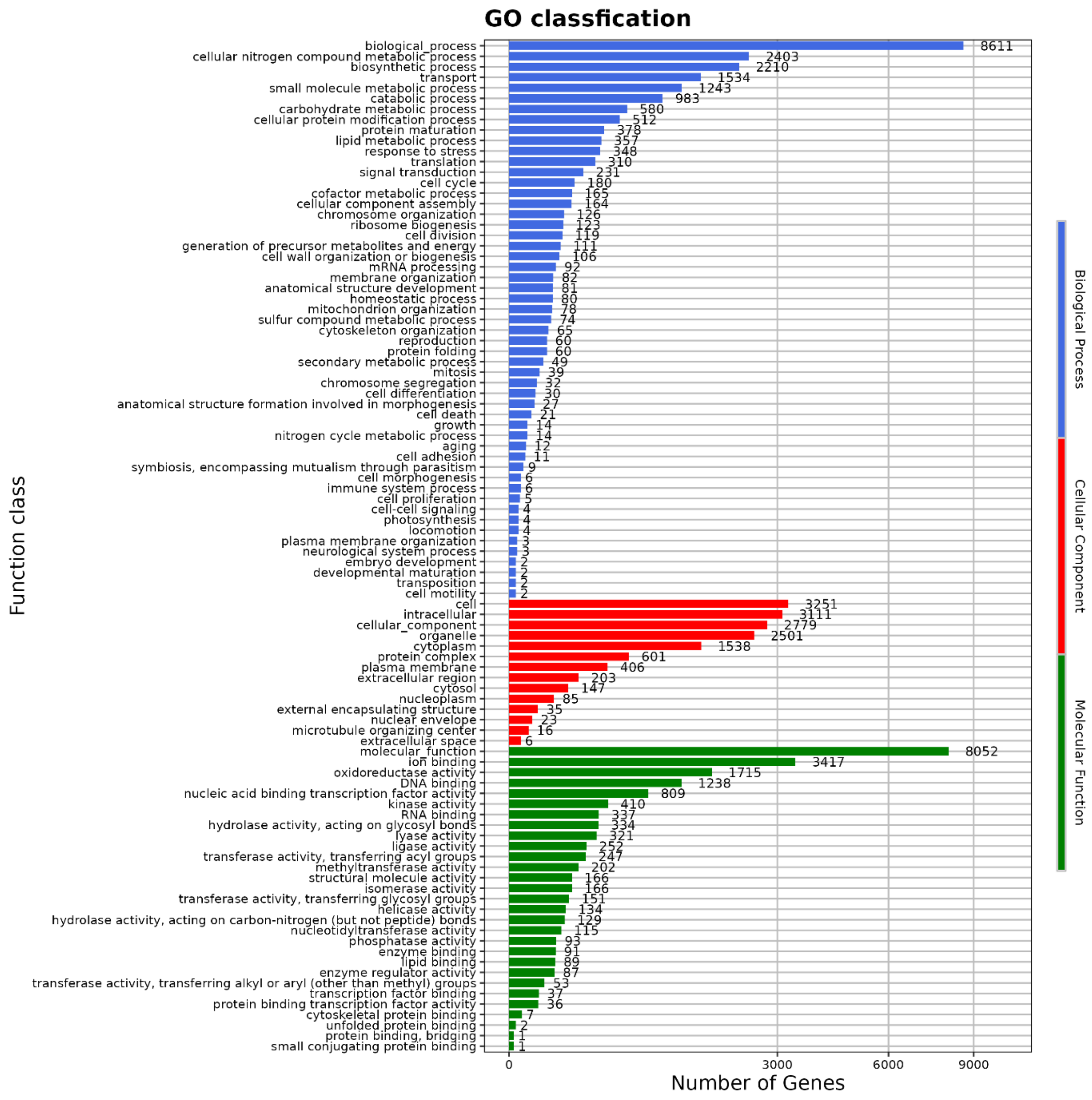

Supplemental Figure S5. GO term classifications for the genome of isolate CNSD1.

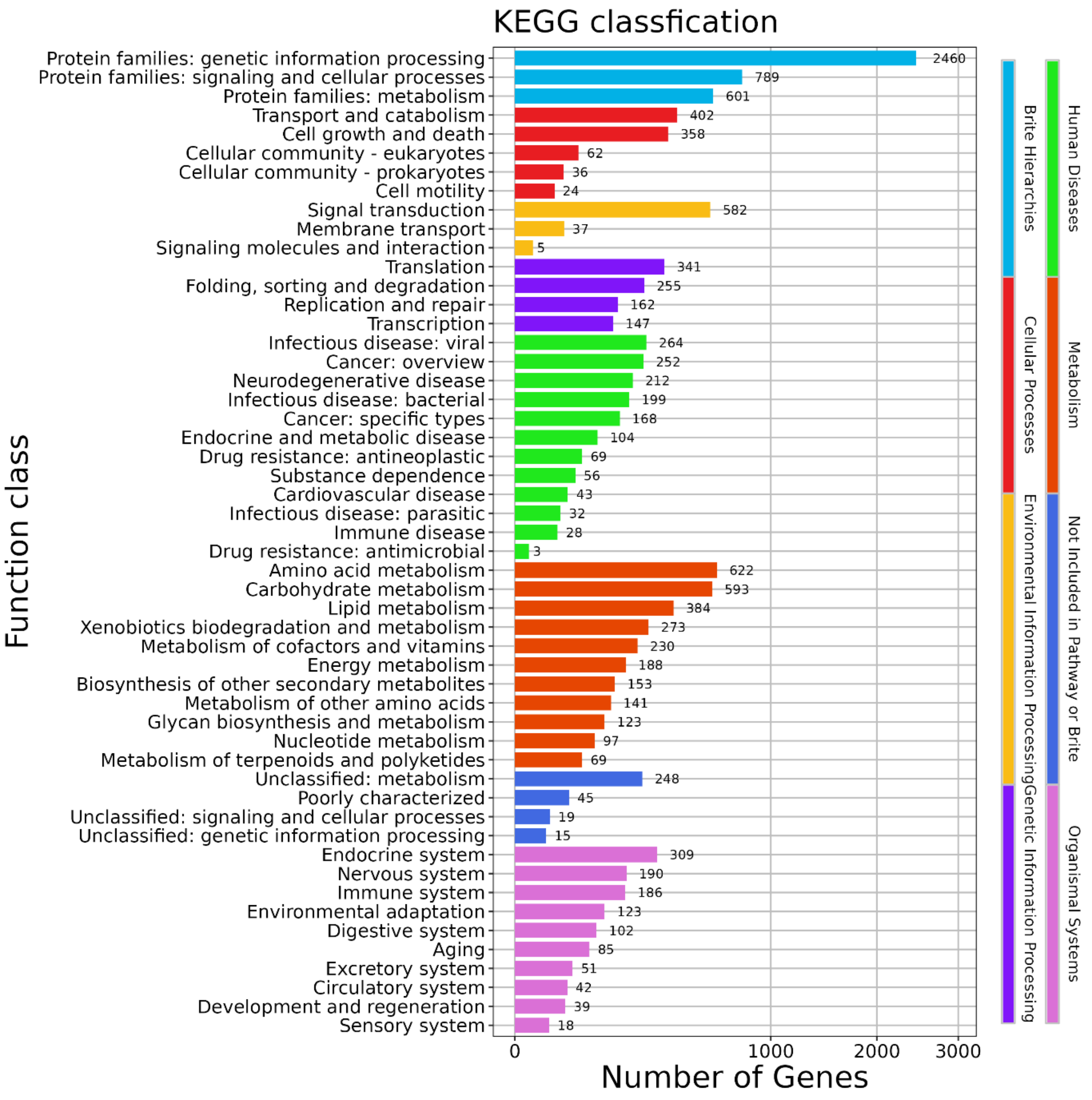

Supplemental Figure S6. KEGG classifications for the genome of isolate 16117.

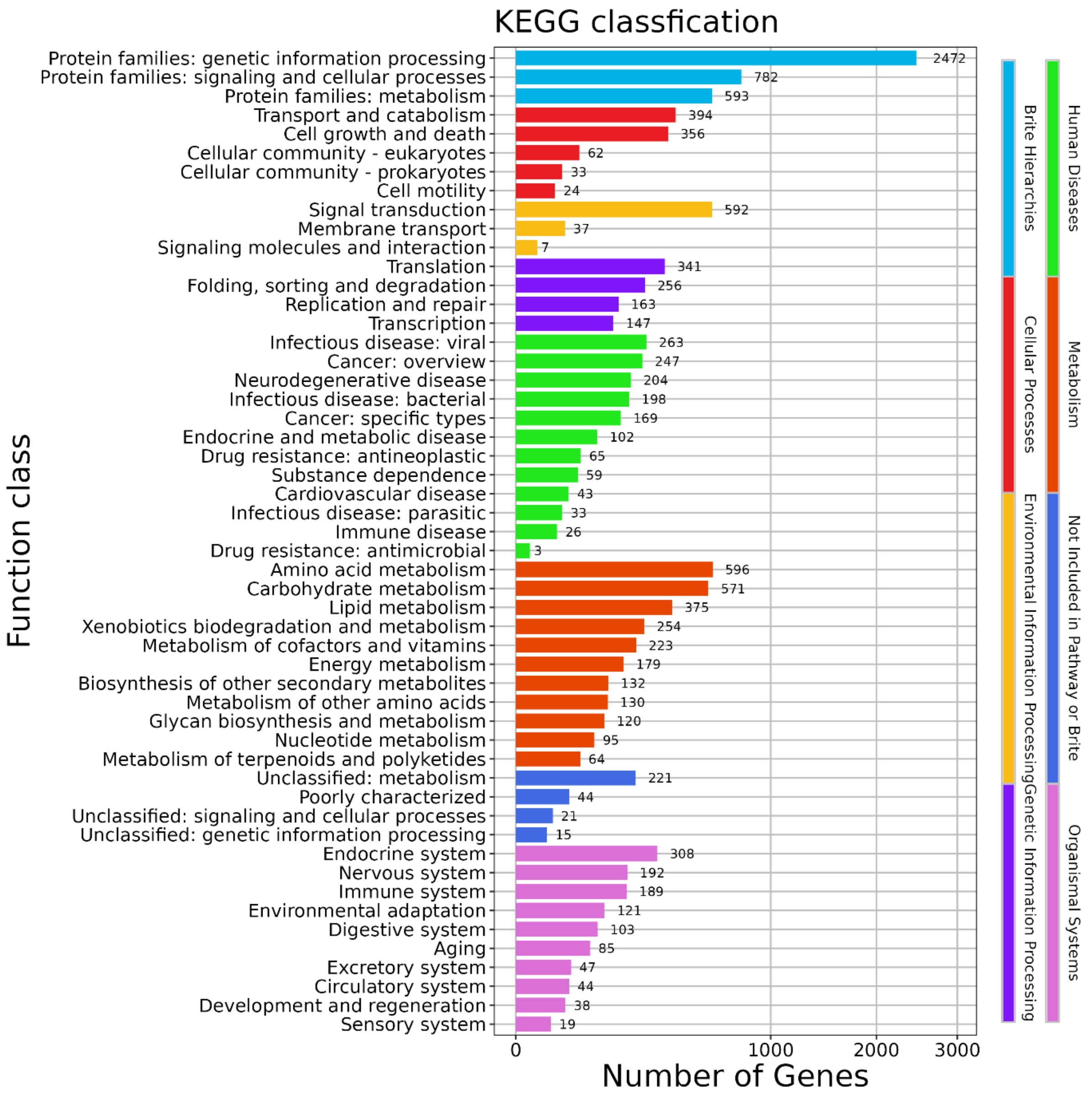

Supplemental Figure S7. KEGG classifications for the genome of isolate CNSD1.

Supplemental Table S1. Banana-derived isolates and their associated genome data or assemblies used in this study.

Isolate name	Assembly or SRA Accession	Source or Institution
Foc410	GCA_000350365.1	Guo et al. 2014
Moz1	GCA_040822185.1	Westerhoven et al. 2024
36102	GCA_040822265.1	Westerhoven et al. 2024
160527	GCA_005930515.1	Asai et a. 2019
TR4	GCA_007994515.1	Warmington et al. 2019
TR4	GCA_021237285.1	Leiva et a. 2022
FocRace4	GCA_027920445.1	Chinese Academy of Tropical Agricultural Sciences
Foc1	GCA_000350345.1	Raman et al. 2021
II5	GCA_031834405.1	Zhang et al. 2024
CR1.1	GCA_040822225.1	Westerhoven et al. 2024

C058	GCA_040822135.1	Westerhoven et al. 2024
C135	GCA_040822145.1	Westerhoven et al. 2024
C081	GCA_040822215.1	Westerhoven et al. 2024
C1HIR-9889	GCA_001696625.1	Raman et al. 2021
VPRI44084	GCA_025216845.1	AgriBio, La Trobe University
VPRI44083	GCA_025216865.1	AgriBio, La Trobe University
VPRI44079	GCA_025216905.1	AgriBio, La Trobe University
VPRI44082	GCA_025216935.1	AgriBio, La Trobe University
VPRI44081	GCA_025216985.1	AgriBio, La Trobe University
FocUH	GCA_034509825.1	Shipman et al. 2023
C2HIR	GCA_045838155.1	Universiti Malaya
C1HIR	GCA_045838205.1	Universiti Malaya
16117_R1	SRR31177457 (PRJNA1178358)	This study
CNDS1_TR4	SRR31177522 (PRJNA1174872)	This study
Bif_04	SRR24907614*	Westerhoven et al. 2024
9_KT06-A1	SRR24907615*	Westerhoven et al. 2024
Phi6,6a	SRR24907616*	Westerhoven et al. 2024
P41b	SRR24907617*	Westerhoven et al. 2024
P26c	SRR24907618*	Westerhoven et al. 2024
P20a	SRR24907619*	Westerhoven et al. 2024
NRRL_36120	SRR24907620*	Westerhoven et al. 2024
56_JB09-A2	SRR24907621*	Westerhoven et al. 2024
NRRL_36118	SRR24907622*	Westerhoven et al. 2024
NRRL_36117	SRR24907623*	Westerhoven et al. 2024
NRRL_36116	SRR24907624*	Westerhoven et al. 2024
NRRL_36115	SRR24907625*	Westerhoven et al. 2024
NRRL_36113	SRR24907626*	Westerhoven et al. 2024
NRRL_36112	SRR24907627*	Westerhoven et al. 2024
NRRL_36110	SRR24907628*	Westerhoven et al. 2024
NRRL_36108	SRR24907629*	Westerhoven et al. 2024
NRRL_36107	SRR24907630*	Westerhoven et al. 2024
NRRL_36103	SRR24907631*	Westerhoven et al. 2024
19_KB07-B	SRR24907632*	Westerhoven et al. 2024
NRRL_36101	SRR24907633*	Westerhoven et al. 2024
Mal43	SRR24907634*	Westerhoven et al. 2024
FocST4-98	SRR24907636*	Westerhoven et al. 2024
FocP1	SRR24907637*	Westerhoven et al. 2024
Foc8	SRR24907638*	Westerhoven et al. 2024
Foc16	SRR24907639*	Westerhoven et al. 2024
F9129	SRR24907640*	Westerhoven et al. 2024
Cub9	SRR24907641*	Westerhoven et al. 2024
15_KTG06-B2	SRR24907643*	Westerhoven et al. 2024
Indo110	SRR25516804*	Westerhoven et al. 2024
C192	SRR25516805*	Westerhoven et al. 2024
C187	SRR25516806*	Westerhoven et al. 2024
C177	SRR25516807*	Westerhoven et al. 2024
C176	SRR25516808*	Westerhoven et al. 2024
C082	SRR25516810*	Westerhoven et al. 2024
Race1	SRR550150*	Guo et al., 2014

CAV2318	CAV2318	Zhang et al. 2024
CAV045	CAV045	Zhang et al. 2024
GD02	GD02	Zhang et al. 2024

These genomes were downloaded from https://mycocosm.jgi.doe.gov/mycocosm/home/releases?flt=fusarium+oxysporum

* Assemblies for these SRR datasets were generated using a pipeline containing Trimmomatic (version 0.36.0) including a ILLUMINACLIP step using Truseq3 adaptors for PE reads, SPAdes assembler (version 3.15.5) with --isolate option, and RepeatMasker (version 4.1.5) using a custom repeat library from RepeatModeler (version 2.0.4, *F. oxysporum*) as the input.

Supplemental Table S2. Clustering of *Fusarium oxysporum* f. sp. *cubense* isolate 16117 proteins based on the functional classification of KOG. The three most abundant categories are highlighted in bold.

KOG categories	categories function	ORF number
A	RNA processing and modification	296
B	Chromatin structure and dynamics	80
C	Energy production and conversion	540
D	Cell cycle control, cell division, chromosome partitioning	122
**E**	**Amino acid transport and metabolism**	**639**
F	Nucleotide transport and metabolism	140
**G**	**Carbohydrate transport and metabolism**	**1121**
H	Coenzyme transport and metabolism	129
I	Lipid transport and metabolism	416
J	Translation, ribosomal structure and biogenesis	410
K	Transcription	446
L	Replication, recombination and repair	284
M	Cell wall/membrane/envelope biogenesis	129
N	Cell motility	2
O	Posttranslational modification, protein turnover, chaperones	621
P	Inorganic ion transport and metabolism	264
**Q**	**Secondary metabolites biosynthesis, transport and catabolism**	**836**
R	General function prediction only	0
S	Function unknown	7315
T	Signal transduction mechanisms	399
U	Intracellular trafficking, secretion, and vesicular transport	365
V	Defense mechanisms	100
W	Extracellular structures	5
Y	Nuclear structure	4
Z	Cytoskeleton	100

Supplemental Table S3. Clustering of *Fusarium oxysporum* f. sp. *cubense* isolate CNSD1 proteins based on the functional classification of KOG. The three most abundant categories are highlighted in bold.

KOG categories	categories function	ORF number
A	RNA processing and modification	302
B	Chromatin structure and dynamics	80
C	Energy production and conversion	506
D	Cell cycle control, cell division, chromosome partitioning	122
**E**	**Amino acid transport and metabolism**	**631**
F	Nucleotide transport and metabolism	132
**G**	**Carbohydrate transport and metabolism**	**1053**
H	Coenzyme transport and metabolism	126
I	Lipid transport and metabolism	400
J	Translation, ribosomal structure and biogenesis	411
K	Transcription	431
L	Replication, recombination and repair	252
M	Cell wall/membrane/envelope biogenesis	122
N	Cell motility	2
O	Posttranslational modification, protein turnover, chaperones	607
P	Inorganic ion transport and metabolism	252
**Q**	**Secondary metabolites biosynthesis, transport and catabolism**	**775**
R	General function prediction only	0
S	Function unknown	7012
T	Signal transduction mechanisms	376
U	Intracellular trafficking, secretion, and vesicular transport	357
V	Defense mechanisms	95
W	Extracellular structures	5
Y	Nuclear structure	4
Z	Cytoskeleton	100

Supplemental Table S4. Classification of *Fusarium oxysporum* f. sp. *cubense* isolate 16117 proteins into the six CAZyme subfamilies.

Class	Genes_Count	Class_Definition
GT	112	Glycosyl Transferases
PL	28	Polysaccharide Lyases
CE	192	Carbohydrate Esterases
AA	146	Auxiliary Activities
CBM	35	Carbohydrate-Binding Modules

GH	392	Glycoside Hydrolases

Supplemental Table S5. Classification of *Fusarium oxysporum* f. sp. *cubense* isolate CNSD1 proteins into the six CAZyme subfamilies.

Class	Genes_Count	Class_Definition
GT	108	Glycosyl Transferases
PL	28	Polysaccharide Lyases
CE	175	Carbohydrate Esterases
AA	136	Auxiliary Activities
CBM	30	Carbohydrate-Binding Modules
GH	363	Glycoside Hydrolases

Supplemental Table S6. Number of effectors in the genomes of *Fusarium oxysporum* f. sp. *cubense* isolates 16117 and CNSD1.

	Number of Genes	
**Program**	**FocR1 16117**	**FocTR4 CNSD1**
SignalP	1643	1551
Tmhmm	3214	3087
Secretory proteins	1357	1276
Subcellular localised proteins (TargetP)	1807	1721
Secretory&SP proteins	1354	1270
Apo/Apo&Cyto effectors (EffectorP)	420/104	417/106
Secreted, SP, Apo/Apo&cyto effectors	265/55	256/57

Supplemental Table S7. SIX gene homologs detected in the genomes of CNSD1 and 16117 using TBLASTN with Fol-Six protein sequences used as queries. %Identity is the percentage identity in pairwise alignment. qstart and qend are the respective start and end of the query sequence. sstart and send are the respective start and end of the scaffold hit sequence.

Isolate	Gene	Scaffold	%Identity	length	qstart	qend	sstart	send	evalue
CNSD1	*SIX1*	ptg000003l	73.90	287	1	284	231,801	232,631	5.5×10^{-136}
CNSD1	*SIX1*	ptg000003l	72.50	284	1	284	707,135	707,947	4.5×10^{-132}
CNSD1	*SIX1*	ptg000003l	70.10	284	1	284	269775	268939	1.4×10^{-129}
CNSD1	*SIX2*	ptg000008l	62.70	233	1	232	6,350,886	6,350,191	4.0×10^{-94}
CNSD1	*SIX4*	ptg000003l	89.00	191	52	242	854,875	854,303	9.4×10^{-102}
CNSD1	*SIX6*	ptg000003l	63.00	208	1	206	856,266	855,643	9.9×10^{-84}
CNSD1	*SIX8*	ptg000003l	77.40	133	24	140	884,769	884,371	3.3×10^{-61}
CNSD1	*SIX8*	ptg000003l	77.40	133	24	140	794,619	795,017	3.3×10^{-61}
CNSD1	*SIX9*	ptg0000077l	53.00	115	1	113	1,035,029	1,034,685	5.3×10^{-31}
CNSD1	*SIX9*	ptg000003l	53.00	115	1	113	63,964	64,308	5.3×10^{-31}

CNSD1	*SIX9*	ptg000003l	45.30	95	14	105	899,469	899,185	2.1×10^{-18}
CNSD1	*SIX13*	ptg000003l	85.90	313	1	293	766,404	767,336	0
CNSD1	*SIX13*	ptg000042l	79.90	313	1	293	25,552	26,490	8.2×10^{-172}
16117	*SIX1*	ptg000019l	71.80	287	1	284	45,472	44,642	2.4×10^{-131}
16117	*SIX1*	ptg000021l	71.80	287	1	284	35,483	34,653	3.5×10^{-131}
16117	*SIX9*	ptg000035l	53.00	115	1	113	94,948	94,604	5.5×10^{-31}
16117	*SIX9*	ptg000030l	53.00	115	1	113	50,422	50,766	5.4×10^{-31}
16117	*SIX13*	ptg000035l	79.90	313	1	293	12,653	13,591	8.6×10^{-172}
16117	*SIX13*	ptg000035l	79.90	313	1	293	156,216	155,278	8.6×10^{-172}
16117	*SIX13*	ptg000005l	79.90	313	1	293	6,042	5,104	8.6×10^{-172}
